# Enhancing research capacity across healthcare and higher education sectors: development and evaluation of an integrated model

**DOI:** 10.1186/1472-6963-12-287

**Published:** 2012-08-28

**Authors:** Anne Whitworth, Shona Haining, Helen Stringer

**Affiliations:** 1 School of Psychology and Speech Pathology, Curtin Health Innovation and Research Institute, Curtin University, GPO Box U1987, Perth, 6845, Australia; 2 NHS North of Tyne, Bevan House, 1 Esh Plaza, Sir Bobby Robson Way, Great Park, Newcastle upon Tyne, NE13 9BA, UK; 3 School of Education, Communication and Language Sciences, Newcastle University, King George VI Building, Queen Victoria Road, Newcastle upon Tyne, NE1 7RU, UK

## Abstract

**Background:**

With current policy in healthcare research, in the United Kingdom and internationally, focused on development of research excellence in individuals and teams, building capacity for implementation and translation of research is paramount among the professionals who use that research in daily practice. The judicious use of research outcomes and evaluation of best evidence and practice in healthcare is integrally linked to the research capacity and capabilities of the workforce. In addition to promoting high quality research, mechanisms for actively enhancing research capacity more generally must be in place to address the complexities that both undermine and facilitate this activity.

**Methods:**

A comprehensive collaborative model for building research capacity in one health professional group, speech and language therapy, was developed in a region within the UK and is presented here. The North East of England and the strong research ethos of this profession in addressing complex interventions offered a fertile context for developing and implementing a model which integrated the healthcare and university sectors. Two key frameworks underpin this model. The first addresses the individual participants’ potential trajectory from research consciousness to research participative to research active. The second embeds a model developed for general practitioners into a broader framework of practice-academic partnership and knowledge and skills exchange, and considers external drivers and impacts on practice and patient outcomes as key elements.

**Results and discussion:**

The integration of practice and academia has been successful in building a culture of research activity within one healthcare profession in a region in the UK and has resulted, to date, in a series of research related outcomes. Understanding the key components of this partnership and the explicit strategies used has driven the implementation of the model and are discussed here.

**Conclusions:**

A strong, equitable collaboration between clinical and academic partners working towards a common outcome can enhance the use of research within the healthcare workforce and contribute actively to the research process. A set of propositions are specified to facilitate both transferability of this partnership model to other professional groups and clinical teams and evaluation of the model components.

## Background

Building sustainable research capacity within the health care professions is fundamental in taking the health research agenda forward and to achieving improved health outcomes [1]. In the UK, this is a key rationale for the establishment of the National Institute for Health Research (http://www.nihr.ac.uk), developed to oversee the conduct of high quality research and committed to ensure capacity for implementation of research outcomes. The need for researchers to be aware of how findings will be used and interpreted by healthcare professionals, and for the research to reflect issues relevant to those at the interface of patient care, are both paramount to successful implementation of research outcomes. To achieve this synergy, all health care professionals need to be actively engaged in the research process in order that they can engage critically with the available evidence [2,3]. This engagement should extend to applying judicious consideration to the recruitment of participants for research programmes, to being able to contribute to the development of research activity by asking practice driven research questions, and to be aware of personal research-readiness in the context of the research process itself. This paper describes a model being used to build this research capacity through ensuring a research-ready workforce that can both implement research outcomes and contribute to further research activity. The key principles underpinning the success of the model are set out and are intended to stimulate debate on the critical factors for enhancing research capacity amongst health care professionals.

### The policy context

The drivers for increased research capacity in the UK workforce have been clearly articulated by Department of Health policy over recent years. In 2006, the Best Research for Best Health [4] strategy led to the creation of the National Institute for Health (NIHR) as a platform for driving and delivering high quality research across the whole National Health Service (NHS). The NIHR sought to achieve this through the development of research-capable staff by funding a Faculty of highly skilled researchers from a range of healthcare professions and by developing the research skills and career paths of future leaders of research through a series of awards and post-graduate training. Funding of a national advice service further assisted professionals wishing to undertake research, in particular, clinical trials, to steer a path through the complex processes involved. This approach was subsequently reinforced through the UK Clinical Research Collaboration (UKCRC) report Developing the Best Research Professionals [5] which presented a framework for developing academic careers, primarily for nurses but also involving allied health professionals. This report focused on the need to develop research training at postgraduate level to produce professionals with the skills to actively develop and lead research activity, and on facilitating opportunities to combine clinical and academic careers. The goal was to “ultimately produce research leaders and academics of the future” (p6) or personnel who would be “capable of operating at the highest levels of research” (p15) [5]. The promotion and conduct of research continues to remain a core NHS role (Equity and Excellence: Liberating the NHS, 2010) [6], with research regarded as integral to increasing the quality and productivity of the NHS; the strategy of promoting research leaders is still at the heart of its activity. What is not as visible within the organization, however, is a systematic focus on the research capabilities of those professionals who need to use the research. Equally, direct input from those same professionals into the research process to facilitate higher levels of research activity in the workplace is not generally sought.

Similar activity around research excellence has been present within the University sector in the UK where the Research Assessment Exercises (RAE) (http://www.rae.ac.uk) and, latterly, the Research Excellence Framework (REF) (http://www.ref.ac.uk) has been set up to evaluate and reward high quality research. While high standards of research are at the heart of these exercises, there is increased attention on the societal impact of University-based research; this has, in turn, become a key driver for universities to collaborate with professional users of research Such a process is directly facilitated by input from practice to ensure the right research is being conducted. Ensuring that this link between the university and healthcare sectors is present in the translational chain is a very real challenge but one regarded as vital if the research agenda is to work in practice. Recent emphasis has been placed on the crucial relationship between local university and healthcare partners to work together to build research capacity. This relationship has been supported by a variety of NIHR funding streams, e.g. Flexibility and Sustainability Funding and the Health Services and Delivery Research programme, to enhance the strategic focus on research.

### Current evidence from models to enhance research capacity

There are no pre-existing models for achieving the cross-sector integration referred to above that focus on the reciprocity of the knowledge and skills required for research. Some of the contributing processes, however, have been highlighted. Atkin et al. [7], in aiming to build research capacity in allied health professionals across one region in the UK, drew on the six phases of Rowan’s [8] research cycle model where professionals undergo a process of identifying necessary research activity from clinical practice, develop and implement a project to address this activity and then disseminate findings back to practice, with the cycle then repeating. Farmer and Weston [9] reported a more systemic model of research capacity-building developed from a general practitioner (GP) and primary health care perspective in an Australian context. Within this model, four categories of GPs were identified in relation to research engagement: (1) non-participants (this made up the largest group) who “have insufficient time or support to undertake research, or even to apply evidence in their clinical practice” (p.1140), (2) those participating in data collection or evaluation of others’ research, (3) those involved in managing research projects, often gaining formal research training, and (4) those academic practitioners who lead on securing funding and supervising teams. Within this model, six guiding principles are required to be in place to facilitate the progression of practitioners from one category to another. These include: (i) a context enabling all GPs from at any stage of the “whole system” to progress between categories, (ii) accommodation of diversity such that the model is responsive to individual backgrounds, interests and learning styles, (iii) reduction of some of the barriers, particularly around paid protected time and peer support, (iv) a collaborative ethos between groups and individuals, potentially through joint academic-clinical posts, (v) access to feedback and mentoring from more experienced researchers and (vi) opportunities for networking.

Farmer and Weston’s concerns around reducing barriers echo other studies. Atkins et al. cited inability to access funding as a significant obstacle in increasing research activity. They proposed that, when specific research funding was difficult to obtain, collaboration between qualified professionals and pre-registration students could provide a fruitful and mutually advantageous environment where professionals could engage with the research process on specific projects. Other barriers relating to managerial and organizational structures have also been clearly documented within healthcare [10,11].

The contribution of each of the components outlined in the above studies is not disputed: the model set out by Farmer and Weston, in particular, provides a useful framework for capturing a range of key principles. These earlier studies, however, arguably do not go far enough in setting out some of the essential components for creating an effective and sustainable research capacity-building environment, in particular in capturing academic-practice partnership. The importance of academic-practice partnership and the focus on reciprocal exchange of knowledge and skills have proven to be core to the activity reported in the current study. This paper outlines a comprehensive model that has been developed and successfully implemented by speech and language therapists in the North East of England. This model, while building overtly on principles akin to those outlined above, develops the principles of academic-practice partnership and knowledge and skills exchange between the two sectors in a reciprocal manner. The professional context for developing this model will be expanded prior to elaboration of the proposed frameworks. Findings of an independent qualitative evaluation [12], commissioned by the partners to explore the impacts of this activity, are also discussed.

### Building a model within a professional context

Health and social care professionals all seek to engage in research-based practice, overseen by the standards of their respective professional colleges and/or councils. While health professionals are the focus of this paper, many issues are common to building research capacity in the social care professions [13,14]. The need to embed, influence and contribute to research is a common driver for each of the professional groups, often linking with local higher education institutions and with funding bodies which commission and/or fund the research. In order to facilitate this activity through enhancing capacity and capability, a well established speech and language therapy partnership in the North East of England involved in collaborative educational practice provided the context for developing a highly productive research collaboration. The establishment of the North of Tyne Speech and Language Therapy Research Collaboration (referred to subsequently as the Collaboration) formalized existing links between Speech and Language Therapy clinical managers in the three Primary Care Trusts within the NHS North of Tyne area, academics in the School of Education, Communication and Language Sciences, Newcastle University, and the Research and Development (R&D) division of the NHS North of Tyne region. Steered by representatives from these organizations, the Collaboration was perceived as an inclusive network whose membership included all speech and language therapists and related academics within the vicinity. With a strong research culture already present within the profession, a model to build research capacity grew organically from the partnership. The components of the model are set out below with a view to this being tested as a transferable framework to different professional groups and to multi-disciplinary groups working in a common clinical area. Further, a set of propositions considered to be critical in embedding research in the workforce are specified.

## Methods

Two key frameworks underpin this model of working, elements of which build on earlier models, but with some important conceptual differences. These revolve around (1) individual participant involvement and (2) practice-academic partnership. These are outlined below, followed by a discussion of the principles involved in the Results and Discussion.

### Individual participant framework

**Figure 1:**
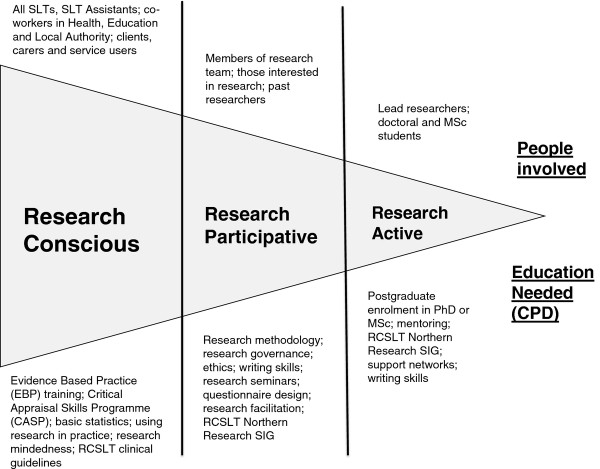
Individual model for mapping professional development needs and research capacity used within the North of Tyne Speech and Language Therapy Research Collaboration.

Identifying the individual participant’s engagement in the research process is essential, recognized in Farmer and Weston’s model in the progression from non-participative to active in research. In the model proposed here, healthcare professionals move from (and between) research conscious to research participative to research active (see Figure 1), a process not dissimilar to making “the transition from research consumer to research facilitator and producer” put forward by Atkin et al. (p. 105) [7]. Unlike Farmer and Weston’s model, however, no category is present for a “non-participating” group of professionals as all members of the speech and language therapy profession are required to engage in research related activity (Health and Care Professions Council (HCPC) Standards of Proficiency; Standard 2b1: be able to use research, reasoning and problem-solving skills to determine appropriate actions; http://www.hcpc-uk.org/assets/documents/10000529Standards_of_Proficiency_SLTs.pdf). Being research conscious is therefore considered an essential level for HCPC-regulated professions working within health and social care in the UK, where individuals have an awareness of research in the workplace and the skills to seek, critique and use evidence already in the public domain as part of their daily practice. This expectation is evidenced in the widespread use of evidence-based clinical guidelines [15] and that, during 2010/11, 97% of Health Trusts in the UK were engaged in portfolio (i.e. open competition) funded research [16]. Furthermore, UK Care Quality Commission regulations on suitability of staffing require a level of knowledge, experience, qualifications and skills which can only be achieved through a workforce being actively engaged with the evidence base (http://www.cqc.org.uk/). To promote inclusiveness, the model identifies co-workers and users as falling within the research aware group while recognizing that formal education proposals set out in the model may not apply and/or would require adaptation. The second level, research participative, is where individuals are involved as a member of a research team or project. Individuals here, often in the context of their clinical team, play a key role in ensuring that research is delivered. Roles such as signposting a patient population for national research projects or gaining consent from relevant prospective participants may be involved here, along with more direct engagement in, for example, carrying out novel complex interventions. Individuals at this level may also be part of a team developing research ideas and projects in collaboration with academics. Those professionals falling into the research active group of individuals include those who are undertaking a research degree at postgraduate level or have research embedded in their substantive job with numerous links to academics and research orientated information and support. Likely members and potential education requirements within each level are set out in Figure 1.

The horizontal cone in the model in Figure 1 broadly reflects the respective proportions of the categories with the majority of healthcare professionals falling within the research conscious group, a smaller proportion participating in research, while a smaller proportion still likely to be active producers/instigators of research. The model is used within the Collaboration to directly increase speech and language therapists’ awareness of their own perceptions of themselves in the research process and enable them to reflect on their own status and trajectory. A fundamental feature here is that all professionals fall somewhere along the research continuum at all times. These roles of engagement in the research process are neither static nor exclusive, with an individual potentially being at different research levels at different times in their careers. Transforming every clinician into an active researcher was not an objective of the Collaboration, a factor important for building confidence in the model; and indeed this would have been counterproductive. Rather, the intention was to embed research awareness within clinical services and into individuals’ own developmental trajectory. This supports the UK NHS Operating Framework (2012/13) that further action is needed to embed a culture that encourages and values research throughout the NHS (http://www.dh.gov.uk/en/Publicationsandstatistics/Publications/PublicationsPolicyAndGuidance/DH_131360). It was also recognized that, in order for all professionals to fall minimally within the research conscious stage of the model and be able to deliver research in practice, planning at an organizational level was necessary to raise awareness of research skills and directly input to skill development. The process, however, of facilitating all professionals within these three levels is supported by a second framework that encompasses the principles of research engagement.

### Practice-academic partnership

**Figure 2:**
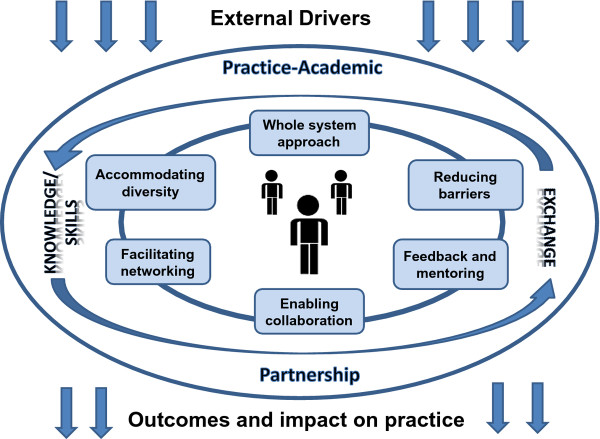
Partnership model for research capacity building used within the North of Tyne Speech and Language Therapy Research Collaboration.

The second framework (see Figure 2) sets out the unique, broader context which is believed to underpin the individual model. This partnership model takes the six areas offered by Farmer and Weston and places them in the context of the wider issues of (i) practice-academic partnership, (ii) knowledge and skills exchange, with emphasis also placed on the importance of identifying both (iii) the external drivers and (iv) the proposed impact on practice and patient outcomes. These issues will be discussed here, while the six areas will be expanded in the following section.

Central to this discussion is the practice-academic partnership which has enabled the reciprocal exchange of knowledge and skills between practitioners and academics from different organizations, and required the reconciliation of different cultures and values. To drive this integrated collaboration, a steering committee involving senior managers from practice (both from the clinical profession and R&D) and from the academic team was formed, and a shared vision was agreed that set out the common values that would underpin the working of the group. Consistent with studies reporting that groups with high levels of value similarity work more effectively [17,18], this process resulted in the development of high levels of trust, transparency and respect amongst the partners [12]. The building of trust, in particular, reported as pivotal to gaining cooperation from partners [19], was central in facilitating effective working within the group.

A second key component is the focus on reciprocity in developing research capacity through a two way exchange of knowledge and skills. Motivated by the clinical issues facing the practitioner, ideas for research that arose directly from practice were systematically solicited and developed through dedicated workshops and events and then linked with the perspectives and skill base of the academic partners. From these events, clinical questions or hypotheses were formulated, aligned to service needs, and appropriate research designs were explored to enable these to be tested. This often required major shifts in perspectives and ownership of ideas, along with a time commitment to the early stages of the process. During this process, it was frequently necessary to clarify the distinction between evidence-based practice and practice-based research, emphasizing the frequent gaps in evidence and the need to create collaboration between clinicians and academics to evaluate new evidence.

A third overarching component of the model is the identification of external drivers underpinning collaborative activity. Understanding the external drivers, often unique to different professional groups, their dependence on the prevailing political, social and economic context, is essential in determining priorities, ensuring motivation, setting up mechanisms for working together, accessing funding and implementing outcomes. Different partners also need to be recognized as having separate drivers. The drivers for the NHS partners in the Collaboration revolved around building a research-ready and -active workforce that would facilitate recruitment into research studies, the translation of research outputs and address the strategic need for an improved evidence base. Additionally, an important motivation for service managers, underpinned by the desire to improve services delivered to patients, was to effect a culture change, i.e. raising the base level of research awareness and skills to a level where staff felt ready and supported to take active steps towards research participation and activity at an appropriate time. Academics were motivated by the advantages of strong links with clinical partners in underpinning robust research outcomes that could be readily embedded in practice. This would enable high levels of clinical relevance to teaching and research programmes within the university and facilitate postgraduate recruitment. Aligning these two cultures more closely was recognized as perhaps the Collaboration’s greatest challenge and, to date, greatest achievement [12]; understanding the underlying differences was important in accommodating them.

Early identification of the proposed outcomes and impact for practice of the collaborative activity is the final overarching factor in the model. While recognizing that some outcomes may be unexpected, these need to be identified and planned for at the outset. These are discussed in more detail later.

## Results and discussion

### Principles of partnership working

With the practice-academic partnership, the drivers and impacts, providing a scaffold to the model, each of the six principles from Farmer and Weston’s model have been incorporated within the framework. These are expanded here and their application discussed in the current professional context. In presenting the model, the individual professional or clinical team is placed at the centre (see Figure 2).

1. Whole system approach A “whole system” approach, discussed by Farmer and Weston, highlights the potential for professionals at different stages of their career to enter the research process, depending on service need, resource capacity, motivation and career path. The model presented here has incorporated this principle, supporting staff at all levels, from new graduates to senior clinical managers, to turn ideas into research projects, and identifying suitable pathways through the research process. Additionally, a unique focus of the activity that has taken place in the North East of England has been the systematic integration of final year students from the Speech and Language Sciences programme at Newcastle University, UK, to engage in activity such as service audit, service evaluation and literature reviews to underpin potential activity. With evidence based practice already firmly bedded within the academic curriculum [20], this work, which forms part of the clinical curriculum each year, has supported pilot studies, grant writing and the emergence of clearer research questions for clinicians in the early stages of forming their ideas. Students have formed an integral part of the activity, enhancing the learning and skill value of the placement, while engaging the student early in the application of research principles in practice. The Collaboration also sought to empower services and organizations, involving both teams and individuals, strategically feeding student projects into service priorities and larger research projects. Examples have included piloting questionnaires for clients in an acute stroke unit, and collecting retrospective client data to inform and develop a prospective data collection protocol for language development norms in children with Down Syndrome.

Collaboration with the Royal College of Speech and Language Therapists (RCSLT) to influence the national agenda [21] has contributed to widening the impact. The Collaboration has hosted national research events and workshops; and contributed directly to policy development through its awareness raising programme.

2. Accommodating diversity Farmer and Weston’s view of accommodating the different research needs of individuals and the diversity of interests and learning styles is closely reflected in this model. While a range of initiatives have aimed to accommodate difference, those that have been most successful have been in targeted but highly inclusive events involving practice and academic partners, delivering workshops through the RCSLT Northern Research Special Interest Group (SIG) and other existing groups, and supporting individuals in one-to-one meetings with academics. The former have included sessions to draw out research questions from practice with a diverse range of populations, linking practitioners directly into opportunities with students or into postgraduate programmes to further develop research skills, where appropriate. Specifically, an MSc in Evidence Based Practice (EBP) in Communication Disorders was established [20] to develop research skills, taking forward small scale projects that strategically address the clinical and service needs of the NHS employing organization to which the student is attached.

3. Reducing barriers Identifying and overcoming barriers are necessary components of all research capacity building activity. In the model discussed here, barriers identified in earlier studies relating to time, funding and organizational structures, were managed through three primary strategies. The first targeted the difficulties raised by access to time and funding through securing small amounts of Flexibility and Sustainability Funding (FSF), a Department of Health funding source available to research active NHS Trusts “that allows for local discretion and management of people to support and develop patient and people driven research” (http://www.nihr.ac.uk). Successful bids for FSF funding were used to support individuals through backfilling time, enabling practitioners to conduct small scale studies, review the literature or prepare larger bids. This resulted in the submission of a succession of bids for NIHR funding. With the practitioner paired with an academic partner, this activity was maximally efficient and supported by an internal bidding process that, through detailed feedback and mentoring, ensured well designed projects. A second strategy was implemented through a further successful FSF bid to fund a Speech and Language Therapy Research Facilitator post (initially for one year). This reduced the barrier of time for the steering group and the academic partners, with the Research Facilitator taking on such roles as the management of events, liaising with Ethics Committees, and navigating different funding streams. The third strategy for overcoming organizational barriers was through the commitment of senior clinical managers within the profession and R&D teams on the Collaboration steering group, establishing a research culture at a high level. This led directly to organizational change through, for example, the explicit labeling of research activities as enhancing research consciousness and the introduction of local team research strategy meetings, each contributing to individuals’ awareness of their position within a research culture. The raised status of journal club activity, for example, was promoted to not only value this as a forum for reading and discussing new research findings, but also as a tool to support research projects through critically appraising relevant literature. The direct proportion of team members participating in research has also been increased through strategic project support from supernumerary resources, e.g. student speech and language therapists.

Interestingly, one barrier identified was a lack of research confidence amongst very able practitioners such that, despite the strategies outlined above, a perception persisted that research both took place away from the workplace (indeed, occurred in ‘ivory towers’ (p.11) [12]) and was beyond the capability of a clinician [12]. Such tasks as conducting literature searches were considered removed from daily clinical practice and both time and support were essential to overcome barriers of this type. A broad based definition of research was therefore regarded as important to enable the workplace to map onto research activity, again legitimizing such activities as audit, journal groups, workplace publications and formal research events within usual practice.

4. Enabling collaborations Farmer and Weston highlighted the role of enabling collaborations in research capacity building, focusing on both intra- and inter-disciplinary collaboration, joint academic-practice appointments and multi-centre projects. This has been a key component in the current model, facilitated through collaborations between students, practitioners and academics, collaborations with industry and the private sector through successful establishment of Knowledge Transfer Partnerships (KTPs) (http://www.ktponline.org.uk/), and collaborations with national research initiatives. Supporting the collaboration between clinicians and academics has also been a focus of the dedicated Research Facilitator position in running joint events and preparing joint research applications.

5. Providing feedback and mentoring Greater access to academic mentoring was proposed by Farmer and Weston as a way of increasing research skills and is embedded within the model proposed here. Both the provision of, and involvement in, research events by academic partners has provided regular input into the discussions related to ideas, research methods and access to funding. Developing ideas through these events has led directly to mentoring of speech and language therapists in planning projects in manageable chunks, often incorporating the use of wider service resources such as journal clubs and student placements. Equally, the pairing up of academic and clinical partners in the robust process of submitting applications for pump- priming of small scale projects has ensured that feedback to clinicians and ongoing mentoring throughout the process has occurred. Where this process has led to the writing of larger bids, this mentoring process has frequently evolved into supervision of formal postgraduate research.

6. Facilitating networking The final component of the model proposed here is that of facilitating networking. Several networking strategies have been employed by the Collaboration. A supportive research environment has been facilitated through linking the Collaboration’s objectives to existing professional groups aimed at continuing professional development and joining up activity where appropriate. The RCSLT Northern Research SIG, in particular, has established itself as a partner of the Collaboration, collaborating in a programmed of skill development and raising research awareness in the workplace. The development of an interactive website, led by the Research Facilitator, to inform, share, educate, and disseminate information has also played a role in facilitation of networks (http://research.ncl.ac.uk/slt/).

### Evaluating outcomes

Tangible outcomes for practice are a key component of the model and, as proposed earlier, clear methods for identifying and measuring these should be stipulated at the outset. Examples of measurable outputs relevant to this work after a five year period of collaboration have included:

1. Funded research activity, either internally or externally funded (see 4 below)

2. Pilot work undertaken to inform larger research projects (e.g. through student projects)

3. Knowledge skills exchange (e.g. several KTPs grants have been awarded)

4. Bids submitted for competitive research funding (e.g. securing of NIHR Research for Patient Benefit funding for a large research project to improve patient outcomes and to inform evidence based commissioning)

5. Ongoing practice evaluation (e.g. regular service evaluations undertaken by supervised students on placement)

6. Research skill development (e.g. skills training by the RCSLT Northern Research SIG, annual events led by the Collaboration to draw out research questions and regularly attended by local clinicians)

7. Post-graduate enrolment of practitioners to both the MSc in Evidence Based Practice in Communication Disorders and doctoral training.

Other objectives of the Collaboration that relate to changing the research culture, increasing research confidence and putting in place accessible processes, are less tangible and have required different measurement instruments. To explore these aspects and add external rigour, an independent evaluation was commissioned by the Collaboration after approximately two years of activity [9]. The evaluation used a qualitative methodology to explore both the perceptions and activities around the work of the group, and the research capacity structures of the associated partners linked to the Collaboration. The empirical basis of this evaluation consisted of semi-structured face-to-face interviews with core members of the Collaboration and key partners, and a focus group comprising speech and language therapy clinicians. Following established principles of qualitative data analysis, the interviews sought to achieve an understanding of the internal structures of the Collaboration and produce a narrative around emergent themes. Evidence of culture change was captured through reported enhancement of research opportunities, a positive influence on job satisfaction and contributing to staff retention. Managers’ encouragement of research activity was viewed as pivotal in shaping a research culture within a service.

### Transferability to other professions and contexts

One area examined by the independent evaluation was the potential for the transferability of the partnership model to other health and social care professionals, with transferability of the principles and processes emerging as a key theme [12]. Despite the contextualized nature of the Collaboration in the North East of England, the model of integrated strategic partnership to build research capacity was considered to be highly relevant to other groups and to other contexts, with the building of strong working relationships being a pivotal component of the partnership model. The Collaboration was seen to have benefitted greatly from its local context, with Newcastle University traditionally having a close relationship with local speech and language therapy services. Additionally, all partners believed that the geographical context provided an environment conducive to innovation and close community ties. The North East of England, due to its history, culture and location, has a strong regional identity; this is less common in England and more akin to that found in the devolved countries of Wales and Scotland. Transferability of the model would therefore depend on an awareness of each local context, considering existing and potential collaborations and distinctive characteristics of a region. This awareness would combine with an understanding of the challenges faced in building integrated partnerships and in developing local strategies. Other components considered central to its success included the perceived long term commitment by all partners, that success should be built slowly and steadily, and that regular review of objectives and strategies needed to be undertaken. Successful transferability would depend on these components being present. The evaluation also identified the need for maximum transparency and mechanisms that facilitated both access and readiness to engage (e.g. being perceived as approachable) as being important components of success for either this or other collaborations.

In order to maximize the success of such a model in building research capacity and facilitate the normalization of research activity in clinical practice, a set of propositions are set out here to guide development, facilitate evaluation and encourage use of the two frameworks in a wider professional health and social care context.

1. When the range of strategic drivers for research at individual and organizational levels are identified and understood at the outset, the partners will be more able to provide a realistic context for identifying achievable, measurable outcomes for practice.

2. The development of a shared vision early in the process based on trust, transparency and inclusivity, and that undergoes regular review, will underpin the success of a group’s activity to a greater degree than if this common ground is not explicit.

3. Equal commitment from both practice and academic partners is necessary to ensure the reciprocal exchange of knowledge and skills.

4. The encouragement of different levels of research engagement will support the development of research activity by accommodating and valuing the diversity of individuals’ interests and career paths.

5. High level support from professional and strategic research managers will facilitate greatest culture change through the legitimization of research practices in the workplace, identification of mechanisms for supporting individuals and/or teams, and the leadership required for sustainability.

6. Regular contact of partners and participants will be facilitated by partners with close geographical proximity and common local drivers, and will draw on existing networks and higher education opportunities to target skill development.

7. Identifying and facilitating access to financial and human resources will enable key barriers to be minimized.

8. When measurable outcomes and impacts for practice are specified at the outset and linked to the strategic aims of the partner organizations, progress towards achieving the goals of an integrated collaboration will be greater than if the strategic link does not exist.

## Conclusion

The increased capability of health and social care professionals to engage with research processes is viewed as fundamental to both the translation of research into practice and to support the broader policy objectives of ensuring excellence in healthcare research. The development of an integrated partnership between health and university sectors in speech and language therapy in the North East of England has accelerated the level of local research activity, with the consequence that research capacity and readiness have increased. While the activity described here is set within one profession and took place in one geographical region in the UK, the principles underpinning the activity are viewed as being relevant and replicable to other health care professions and clinical teams in other locations. Propositions which lie at the heart of the Collaboration described here signpost the way for enhancing research capacity in healthcare with a view to improved patient outcomes.

## Endnotes

^a^At the time of press, this funding stream was titled Research Capability Funding.

## Abbreviations

NHS: National Health Service; NIHR: National Institute for Health Research; R&D: Research and Development; SIG: Special Interest Group; RCSLT: Royal College of Speech and Language Therapists; SLT: Speech and Language Therapist; EBP: Evidence Based Practice; KTP: Knowledge Transfer Partnership; FSF: Flexibility and Sustainability funding; NMAHPs: Nursing, Midwifery and Allied Health Professions.

## Competing interests

The author(s) declare that they have no competing interests.

## Authors’ contributions

All authors contributed equally. All authors read and approved the final manuscript.

## Pre-publication history

The pre-publication history for this paper can be accessed here:

http://www.biomedcentral.com/1472-6963/12/287/prepub
